# Association between Early Childhood Vitamin D Status and Age 6-Year Lung Function among Children with a History of Severe Bronchiolitis in Infancy

**DOI:** 10.3390/nu15102379

**Published:** 2023-05-19

**Authors:** George Doumat, Geneva D. Mehta, Jonathan M. Mansbach, Kohei Hasegawa, Carlos A. Camargo

**Affiliations:** 1 Emergency Medicine Network, Massachusetts General Hospital, 125 Nashua Street, Suite 920, Boston, MA 02114, USA; 2 Division of Allergy and Clinical Immunology, Brigham and Women’s Hospital, Boston, MA 02115, USA; 3 Division of General Pediatrics, Boston Children’s Hospital, Boston, MA 02115, USA

**Keywords:** vitamin D, serum 25-hydroxyvitamin D, lung function, spirometry, bronchiolitis

## Abstract

Improving lung health in children requires understanding the risk factors for decreased lung function. Our objective was to investigate the association between serum 25-hydroxyvitamin D (25(OH)D) levels and lung function in children. We analyzed data from a prospective cohort of infants hospitalized with bronchiolitis (severe bronchiolitis), a group at high risk for developing childhood asthma. Children were followed longitudinally, and 25(OH)D and spirometry testing were conducted at ages 3 and 6, respectively. We used a multivariable linear regression adjusted for race/ethnicity, annual household income, premature birth, and secondhand smoke exposure to examine the association between serum 25(OH)D level and primary outcomes (percent predicted [pp] of forced expiratory volume in the first second (FEV1) and the forced vital capacity (FVC)) and secondary outcome (FEV1pp/FVCpp). Serum 25(OH)D level and age 6 spirometry were available for 363 children. In adjusted analyses comparing the highest quintile (Q5) of serum 25(OH)D (median 37 ng/mL) to the lowest quintile (Q1; median 18 ng/mL), FEV1pp was 6% lower (*p* = 0.03) in Q1. Likewise, FVCpp was 7% lower (*p* = 0.03) in Q1. There was no difference in FEV1pp/FVCpp across the serum 25(OH)D quintiles. Compared to children with higher vitamin D status at age 3, those with lower status had decreased FEV1pp and FVCpp at 6 years.

## 1. Introduction

Vitamin D deficiency is a common problem in children, affecting approximately 10% to 12% of children [1,2]. The effect of vitamin D deficiency on rickets and osteomalacia is well established, but its effect on non-bone-related disorders is less clear [3]. Animal studies have shown that vitamin D deficiency causes airway remodeling, resulting in structural changes that affect lung function [4,5], but the association in humans remains uncertain.

Observational studies conducted in the general population showed no association between plasma 25-hydroxyvitamin D (25[OH]D) levels, the best available marker of vitamin D status, and lung function [6]. On the other hand, a study on the general community demonstrated a dose–response relationship between insufficient serum 25(OH)D levels and worse lung function in children [7]. Among children with asthma or cystic fibrosis, studies have shown mixed results [8,9,10,11]. No studies have examined the association between early childhood vitamin D status and age 6 lung function among children who were hospitalized for bronchiolitis in infancy (severe bronchiolitis). Bronchiolitis is the most common cause of infant hospitalization in the United States (US) [12], and also a major risk factor for childhood asthma [13].

Given the inconsistency of prior studies in the scientific literature, and the specific lack of studies on infants with severe bronchiolitis, our objective was to investigate the association between early childhood serum 25(OH)D level and subsequent lung function among children with a history of severe bronchiolitis in infancy. We hypothesized that children with lower serum 25(OH)D levels at age 3 have decreased lung function at age 6 compared to children with higher levels.

## 2. Materials and Methods

### 2.1. Study Design, Setting, and Participants

We analyzed data from an ongoing, prospective cohort study of infants enrolled at less than 12 months of age. The 35th Multicenter Airway Research Collaboration (MARC-35) cohort is comprised of infants hospitalized with an attending physician’s diagnosis of bronchiolitis (severe bronchiolitis) during 1 of 3 consecutive bronchiolitis seasons from November through April (2011–2014) at one of 17 participating sites across 14 US states. Enrolling physicians were asked to use the American Academy of Pediatrics (AAP) guidelines’ definition of bronchiolitis—i.e., an acute respiratory illness with some combination of rhinitis, cough, tachypnea, wheezing, crackles, and retractions [14]. We excluded children with known heart or lung disease, immunodeficiency, immunosuppression, or gestational age < 32 weeks.

Of 921 children in the MARC-35 cohort, 373 (41%) completed both the age 3 visit for serum 25(OH)D testing and the age 6 visit for spirometry. The criteria for excluding individuals from the spirometry test involved using bronchodilators before the test. This included using short-acting beta-agonists (SABA) within 4–6 h, short-acting muscarinic antagonists (SAMA) within 12 h, long-acting beta-agonists (LABA) within 24 h, and long-acting muscarinic antagonists (LAMA) within 36–48 h. The institutional review board at each participating hospital approved the study. Written informed consent was obtained from the parent or guardian, and assent for spirometry was obtained from the child.

### 2.2. Data Collection

During the enrollment process, we conducted a structured interview to evaluate participants’ demographic features, as well as their medical, environmental, and family history. Subsequently, we conducted parental interviews every six months via telephone.

We measured serum 25(OH)D levels at age 3 and spirometry at age 6. Blood samples were collected and stored at −80 °C until serum 25(OH)D levels were measured. Serum 25(OH)D was measured using liquid chromatography–tandem mass spectrometry, with a lowest reportable value of 1 pg/mL [15]. The spirometry was collected in pediatric pulmonary function labs by respiratory therapists trained in accordance with the guidelines set forth by the American Thoracic Society (ATS) and European Respiratory Society (ERS) [16]. Spirometry was then reviewed by a study physician trained in spirometry interpretation for acceptability and reproducibility as per 2019 ATS/ERS criteria [17]. All data were reviewed at the EMNet Coordinating Center at the Massachusetts General Hospital (Boston, MA, USA), and site investigators were queried about missing data and discrepancies identified during manual data checks.

### 2.3. Exposure and Outcome Measures

The primary exposure was age 3 serum 25(OH)D level. Serum 25(OH)D level was analyzed as a categorical ordinal variable (quintiles) [18,19] and as a continuous variable (per 1 ng/mL increment).

The primary outcomes were percent-predicted (pp) values for the forced expiratory volume in the first second (FEV1) and the forced vital capacity (FVC). The secondary outcome was FEV1pp/FVCpp. Sites provided absolute values of all lung function measurements, and we converted these into sex-, age-, and height-adjusted pp values based on Global Lung Initiative (GLI) reference equations [20]. Since a growing body of evidence encourages the move away from using race correction from reference equations, we analyzed with a race-neutral equation by coding race/ethnicity as “other” for all participants [21]. For comparison with the extant literature, we also repeated the analysis using the GLI reference equations that incorporate race.

### 2.4. Statistical Analysis

To adjust for seasonal variations in serum 25(OH)D level, we used a sinusoidal model to calculate de-seasonalized serum 25(OH)D levels [22]. This involved determining the midpoint between the highest and lowest levels of 25(OH)D that occur throughout the year, based on the individual’s baseline 25(OH)D level and the date the individual’s blood was collected.

We used the independent sample *t*-test, ꭓ^2^, or the Wilcoxon rank–sum tests, as appropriate, to examine the inter-group participant characteristics. We used multivariable linear regression modeling to assess the association between age 3 serum 25(OH)D level and FEV1pp, FVCpp, and FEV1pp/FVCpp at 6 years of age. Spirometry values were already “adjusted” for age, sex, and height by using GLI reference equations [20]. The models were further adjusted for race/ethnicity (non-Hispanic White, non-Hispanic Black, Hispanic, and Other); annual household income (socioeconomic status), categorized as less than $40,000, $40,000 to $79,999, and $80,000 or more; premature birth (≤37 weeks); and secondhand smoke exposure. The analyses were repeated using GLI equations that incorporate race and with maneuvers meeting ATS criteria for both acceptability and reproducibility. A two-sided *p* < 0.05 was considered statistically significant. All analyses were performed using Stata 15.1 (Stata Corp, College Station, TX, USA).

## 3. Results

### 3.1. Participant Characteristics

Of the 373 children who completed age 3 serum 25(OH)D testing and age 6 spirometry testing, 6 were excluded due to the inability to produce at least one acceptable maneuver despite adequate coaching, and 4 due to the use of a bronchodilator within 24 h of the spirometry visit, resulting in 363 participants who comprised our analytic cohort. Of these, 288 (79%) produced acceptable and reproducible spirometry per the ATS/ERS criteria, while 75 (21%) produced one or more acceptable maneuvers but did not demonstrate reproducibility.

There was no significant difference in terms of age at enrollment, sex, race/ethnicity, annual household income, premature birth, and secondhand smoke exposure between the 363 participants who completed age 3 serum 25(OH)D testing and age 6 spirometry testing (the analytical cohort), and the 558 participants who did not (the non-analytical cohort; Supplement Table S1).

In the analytic cohort, the median serum 25(OH)D level was 26.5 ng/mL with a range of 8.4–53.0 ng/mL (Table 1). The average age of spirometry was 6.9 years, with no differences among quintiles. There were no significant differences among quintiles in the proportion of males, premature birth, and secondhand smoke exposure. Participants in the lowest quintile of serum 25(OH)D were more likely to be non-Hispanic Black, participants in the second quintile were more likely to be Hispanic, and participants in the top three quintiles were more likely to be White. When assessing the yearly household income, participants in the lower two 25(OH)D quintiles were more likely to live in households making less than $40,000 per year, compared to those in the highest two quintiles, who were more likely to live in households making more than $80,000 per year.

### 3.2. Age 3 Serum 25(OH)D Level and Age 6 Lung Function

The median values for lung function testing were normal (FEV1 = 1.4 L, FEV1pp = 106, FVC = 1.6 L, FVCpp = 109, FEV1/FVC = 0.9, and FEV1pp/FVCpp = 96) (Table 2).

#### 3.2.1. Associations across Quintiles of Serum 25(OH)D

The unadjusted analysis showed a statistically significant association between the quintiles of serum 25(OH) D level and lung function (Table 3). Compared to children in the highest quintile (Q5), those in the lowest quintile had an 11% lower FEV1 (β = −11.0; 95% CI: −16.1, 6.0; *p* ≤ 0.001) and 12% lower FVC (β = −11.8; 95% CI: (−17.1, −6.5; *p* ≤ 0.001). There was no association between serum 25(OH)D quintiles and the FEV1pp/FVCpp.

The adjusted analysis showed a statistically significant association between quintiles of serum 25(OH)D and lung function (Table 4). Compared to children in the highest quintile (Q5), those in the lowest quintile had a 6% lower FEV1pp (β = −6.2; 95% CI: −11.7, −0.7; *p* = 0.03), and those in the fourth quintile (Q4) had a 5% lower FEV1pp (β = −5.3; 95% CI: −9.9, −0.6; *p* = 0.03). Those in the lowest quintile also had a 7% lower FVCpp (β = −6.8; 95% CI: −12.8, −0.9; *p* = 0.03) than those in the highest quintile. There was no significant association between serum 25(OH)D quintiles and the FEV1pp/FVCpp.

The findings persisted when the analysis was repeated using GLI reference equations that incorporate race (Supplement Table S2) and when we restricted the analysis to children who met the ATS criteria for both acceptability and reproducibility (Supplement Table S3).

#### 3.2.2. Associations for Continuous Serum 25(OH)D

The unadjusted analysis showed no association between the continuous serum 25(OH)D level (per 1 ng/mL increment) and FEV1pp (β = 0.2; 95% CI: −0.1, 0.4; *p* = 0.15), FVCpp (β = 0.1; 95% CI: −0.1, 0.4; *p* = 0.19), or FEV1pp/FVCpp (β = 0.02; 95% CI: −0.1, 0.1; *p* = 0.74).

The adjusted analysis showed a non-significant association between serum 25(OH)D level and FEV1pp (β = 0.2; 95% CI: −0.04, 0.5; *p* = 0.05). The adjusted analysis also showed a statistically significant association between continuous serum 25(OH)D and FVCpp (β = 0.3; 95% CI: 0.02, 0.5; *p* = 0.048) but, again, no association between serum 25(OH)D and FEV1pp/FVCpp (β = −0.01; 95% CI: −0.2, 0.1; *p* = 0.90).

## 4. Discussion

We investigated the association between age 3 vitamin D status, as measured by serum 25(OH)D level, and age 6 lung function among a large prospective cohort of children hospitalized with bronchiolitis in infancy. We found that children in the lowest quintile of serum 25(OH)D had lower FEV1 and FVC than those in the highest quintile.

There are few studies on this topic, even among all children from the general population. For example, a study in Taiwan examined the association between serum 25(OH)D levels and lung function in a cohort of 1315 children aged 5–18 [7]. The study showed that children with insufficient (20–29.9 ng/mL) or deficient (<20 ng/mL) serum 25(OH)D levels had lower FVC and FEV1 compared to children with sufficient levels (≥30 ng/mL). This relationship was dose-dependent since children with deficient serum 25(OH)D levels had even lower FEV1 and FVC than children with insufficient levels. Although the Taiwanese children were older and from the general population, these results lend support to our findings since our data showed 6% lower FEV1pp and 7% lower FVCpp among the children in the lowest quintile of serum 25(OH)D compared to those in the highest quintile. However, the middle quintile did not show an association. This may be due to lower sample size, differences in participants’ age, and the differences in reported lung function parameters (percent predicted in our study).

Other studies have examined the association of serum 25(OH)D levels with lung function at later ages. For example, 2607 adolescents underwent 25(OH)D level testing at the age of 10 and spirometry at the age of 15 [23]. The study showed that those with higher 25(OH)D levels had higher FEV1 and FVC, and a lower FEV1/FVC ratio. On the other hand, a clinical trial supplemented 442 adults with vitamin D for 1.1 years and assessed lung function via spirometry afterward. The study showed that vitamin D supplementation did not improve lung function for everyone, but benefited participants who ever smoked (*n* = 217), especially those with asthma/COPD (*n* = 60) or vitamin D deficiency (*n* = 54) [24]. The differences in results at different ages (from toddlers to older adults) may be explained by the potential role vitamin D has on lung function during the postnatal lung development period that can continue until early adulthood [25] and by the decline in lung function with age [26].

Our study population was children with a history of severe bronchiolitis in infancy, which is associated with the risk of developing wheeze and childhood asthma [27]. Three studies examined the association between vitamin D and lung function in children (ages 6 to 16 years) with asthma. Two trials (with 176 and 29 participants, respectively) showed no significant association between vitamin D supplementation and FEV1, FVC, or FEV1/FVC [8,9]. A third trial explored the effect of vitamin D on lung function assessed by forced oscillation technique in asthmatic children. The trial included 84 children aged 3–18 years and showed no association between vitamin D supplementation and lung function measured as percent predicted of forced oscillation technique measures [10]. The variation in findings between the trials and our results might be ascribed to the influence of asthma control and medications on pulmonary function, independent of the impact of serum 25(OH)D. Additionally, the trials excluded children with very low serum 25(OH)D levels, which can affect the results since studies have shown that the relationship between serum 25(OH)D and lung function could be dose dependent [7]. Indeed, the differences we observed were between the highest and lowest quintiles of serum 25(OH)D. The differences can also be attributed to differences in vitamin D supplementation and serum 25(OH)D levels since they are not directly comparable and short-term supplementation is not the same as high levels of serum 25(OH)D. Additionally, the trials’ findings depend on dose, frequency, and duration of supplementation. Finally, the difference in results might be due to the lower sample size in these three trials.

The pathophysiologic mechanisms underlying the association between serum 25(OH)D and lung function are not clear. However, there are proposed mechanisms to explain the association. For example, a study using mouse models showed that vitamin D deficiency causes decreased lung volume, leading to deficits in lung function [4]. Vitamin D deficiency did not alter the architecture of the lung; however, it decreased lung volume by changing lung size. Another proposed mechanism of the effect of low vitamin D status on lung development involves the downregulation of TGF-β1 and TGF-β receptor I during early lung development, which may contribute to airway remodeling and hyperresponsiveness [5]. Moreover, evidence shows that vitamin D exerts immunomodulatory effects by suppressing inappropriate Th1 to environmental exposure (i.e., allergens, infection load). This leads to a more balanced immune response, inhibiting allergic diseases and autoimmune diseases [28]. Thus, vitamin D deficiency can affect lung function by decreasing lung volume, causing airway remodeling, and exerting an immunomodulatory effect.

It is important to explore the association between low serum 25(OH)D and lung function in childhood because it may predispose children to an increased risk of pulmonary disease later in their lives. Low serum 25(OH)D levels are associated with an increased risk of acute respiratory infections [29], asthma exacerbation [30], and chronic obstructive lung disease (COPD) exacerbation [31].

In designing our study, we chose to run the analysis using both race-neutral GLI reference equations and GLI reference equations that incorporate race. Although there is currently no consensus on the optimal method for eliminating race-based adjustments from spirometry results, we recognize the significance of acknowledging the use of race as a sociopolitical construct in medicine to account for individual variations that may inadvertently reinforce health inequalities and structural racism. Therefore, we have taken steps to address this issue by presenting our results using both methods [21].

The study has potential limitations. First, only 41% of the cohort children participated in a serum 25(OH)D testing and spirometry visit. Although this rate is consistent with similar studies (25%) [7], it may result in non-response and attrition bias. Although we assessed major factors that potentially influence vitamin D status and lung function, and observed no differences between the analytical and non-analytical cohorts (Supplement Table S1), we did not assess daily physical activity. Second, this study did not include children with bronchiolitis who did not require hospitalization. Consequently, generalizability to all children with a more general “history of bronchiolitis” merits further investigation. Additionally, serum 25(OH)D was measured only at a single time point (3-year exam), and additional measurements closer to the spirometry testing date would offer a better understanding of the association. Another limitation is the potential confounding effect of socioeconomic status and air pollution on the results. Moreover, our cohort lacks participants with very low serum 25(OH)D levels (e.g., <12 ng/mL); the median 25(OH)D in the lowest quintile was 17 ng/mL. Therefore, we could not assess the associations for very low vitamin D status. Lastly, we included in our analysis children who met the acceptability but not reproducibility criteria as per ATS/ERS guidelines because of the young age of the children. However, the results were unchanged when we restricted the analysis to include the children who met both criteria (Supplement Table S3).

## 5. Conclusions

Among children with severe bronchiolitis in infancy, lower serum 25(OH)D levels at age 3 were associated with decreased FEV1pp and FVCpp at age 6. Our observations should facilitate further investigations into the role of vitamin D in lung development and lifelong lung health.

## Figures and Tables

**Table 1:** Participant characteristics across quintiles of 25(OH)D.

	Q1 (*n* = 73) (8.4–20.1 ng/mL)	Q2 (*n* = 73) (20.2–24.2 ng/mL)	Q3 (*n* = 72) (24.3–27.7 ng/mL)	Q4 (*n* = 73) (27.8–31.8 ng/mL)	Q5 (*n* = 72) (31.9–53.0 ng/mL)	*p*-Value	Total (*n* = 363)
Serum 25(OH)D in ng/mL, median (IQR)	17.8 (16.0–19.0)	22.5 (21.5–23.5)	26.2 (25.2–26.9)	29.5 (28.6–30.7)	35.7 (33.5–39.7)	---	26.5 (21.8–31.2)
Age at spirometry in years, mean (SD)	6.9 (0.7)	6.9 (0.6)	7.1 (0.9)	6.9 (0.6)	6.9 (0.7)	0.06 *	6.9 (0.71)
Male sex, *n* (%)	35 (53)	50 (69)	39 (58)	52 (66)	49 (63)	0.35 †	225 (62)
Race/ethnicity, *n* (%)						**<0.001** †	
Non-Hispanic White	11 (17)	21 (29)	34 (51)	44 (56)	56 (72)		166 (46)
Non-Hispanic Black	31 (47)	22 (30)	14 (21)	12 (15)	6 (8)		85 (23)
Hispanic	23 (35)	30 (41)	15 (22)	19 (24)	15 (19)		102 (28)
Other	1 (2)	0	4 (6)	4 (5)	1 (1)		10 (3)
Yearly household income, *n* = 262 (%)						**0.01** †	
≥$80,000	8 (20)	10 (22)	14 (27)	31 (49)	25 (41)		88 (34)
$40,000–$79,999	10 (24)	13 (29)	16 (31)	10 (16)	18 (30)		67 (26)
<$40,000	23 (56)	22 (49)	22 (42)	22 (35)	18 (30)		107 (41)
Premature birth (<37 weeks), *n* (%)	14 (21)	16 (22)	8 (12)	14 (18)	19 (24)	0.39 †	71 (20)
Secondhand smoke exposure, *n* (%)	11 (17)	11 (15)	8 (12)	7 (9)	10 (13)	0.78 †	47 (13)

Abbreviations: IQR, interquartile range; SD, standard deviation; 25(OH)D, 25-hydroxyvitamin D; Q1, first (lowest) quintile of serum 25(OH)D; Q2, second quintile of serum 25(OH)D; Q3, third quintile of serum 25(OH)D; Q4, fourth quintile of serum 25(OH)D; Q5, fifth (highest) quintile of serum 25(OH)D. * ANOVA (analysis of variance) test † Chi-squared test. Bold denotes *p* < 0.05.

**Table 2:** Age 6 lung function across quintiles of age 3 serum 25(OH)D.

	Q1 (*n* = 73) (8.4–20.1 ng/mL)	Q2 (*n* = 73) (20.2–24.2 ng/mL)	Q3 (*n* = 72) (24.3–27.7 ng/mL)	Q4 (*n* = 73) (27.8–31.8 ng/mL)	Q5 (*n* = 72) (31.9–53.0 ng/mL)	Total (*n* = 363)
FEV1 (L)	1.3 (1.1, 1.5)	1.4 (1.2, 1.5)	1.3 (1.1, 1.6)	1.3 (1.2, 1.5)	1.5 (1.3, 1.6)	1.4 (1.2, 1.6)
FEV1 percent predicted	98 (88, 113)	102 (91, 111)	106 (94, 118)	105 (97, 112)	113 (102, 117)	106 (94, 115)
FVC (L)	1.5 (1.3, 1.7)	1.6 (1.3, 1.8)	1.6 (1.4, 1.8)	1.6 (1.4, 1.8)	1.7 (1.5, 1.8)	1.6 (1.4, 1.8)
FVC percent predicted	104 (89, 115)	104 (90, 120)	109 (100, 117)	110 (102, 119)	115 (106, 123)	109 (98, 119)
FEV1/FVC	0.87 (0.84, 0.91)	0.87 (0.82, 0.93)	0.87 (0.81, 0.91)	0.86 (0.81, 0.91)	0.88 (0.84, 0.92)	0.87 (0.83, 0.91)
FEV1 percent predicted/FVC percent predicted	96 (92, 100)	95 (92, 101)	96 (92, 100)	94 (90, 99)	98 (92, 101)	96 (92, 100)

Values are median and interquartile range (IQR) Abbreviations: L: Liters; 25(OH)D, 25-hydroxyvitamin D; Q1, first (lowest) quintile of serum 25(OH)D; Q2, second quintile of serum 25(OH)D; Q3, third quintile of serum 25(OH)D; Q4, fourth quintile of serum 25(OH)D; Q5, fifth (highest) quintile of serum 25(OH)D.

**Table 3:** Unadjusted association between age 3 serum 25(OH)D and age 6 lung function.

	FEV1 Percent Predicted		FVC Percent Predicted		FEV1 Percent Predicted/FVC Percent Predicted	
Serum 25(OH)D Quintiles	Beta Coefficient (95% CI)	*p*-Value	Beta Coefficient (95% CI)	*p*-Value	Beta Coefficient (95% CI)	*p*-Value
Q5 (31.9–53.0 ng/mL)	Reference					
Q4 (27.8–31.8 ng/mL)	−6.3 (−11.2, −1.5)	**0.01**	−4.6 (−9.6, 0.5)	0.08	−1.7 (−4.1, 0.7)	0.15
Q3 (24.3–27.7 ng/mL)	−5.7 (−10.8, −0.6)	**0.03**	−5.5 (−10.8, −0.2)	**0.04**	−0.5 (−3.0, 1.9)	0.67
Q2 (20.2–24.2 ng/mL)	−9.2 (−14.1, −4.2)	**<0.001**	−9.0 (−14.2, −3.8)	**0.001**	−0.3 (−2.7, 2.2)	0.84
Q1 (8.4–20.1 ng/mL)	−11.0 (−16.1, −6.0)	**<0.001**	−11.8 (−17.1, −6.5)	**<0.001**	−0.01 (−2.5, 2.5)	0.99

Abbreviations: 25(OH)D, 25-hydroxyvitamin D; Q1, first (lowest) quintile of serum 25(OH)D; Q2, second quintile of serum 25(OH)D; Q3, third quintile of serum 25(OH)D; Q4, fourth quintile of serum 25(OH)D; Q5, fifth (highest) quintile of serum 25(OH)D. Bold denotes *p* < 0.05.

**Table 4:** Adjusted association between quintiles of early childhood serum 25(OH)D and age 6 lung function.

	FEV1 Percent Predicted		FVC Percent Predicted		FEV1 Percent Predicted/FVC Percent Predicted	
	Beta Coefficient (95% CI)	*p*-Value	Beta Coefficient (95% CI)	*p*-Value	Beta Coefficient (95% CI)	*p*-Value
Serum 25(OH)D						
Q5	Reference					
Q4	−5.3 (−9.9, −0.6)	**0.03**	−4.4 (−9.4, 0.7)	0.09	−0.9 (−3.6, 1.9)	0.55
Q3	−3.6 (−8.5, 1.4)	0.16	−4.3 (−9.7, 1.0)	0.11	0.4 (−2.6, 3.4)	0.79
Q2	−5.1 (−10.4, 0.1)	0.06	−5.3 (−11.0, 0.4)	0.07	0.04 (−3.1, 3.2)	0.98
Q1	−6.2(−11.7, −0.7)	**0.03**	−6.8 (−12.8, −0.9)	**0.03**	0.4 (−2.9, 3.7)	0.83
Race/ethnicity						
White	Reference					
Black	−13.5 (−18.3, −8.8)	**<0.001**	−14.0 (−19.1, −8.8)	**<0.001**	−0.7 (−3.5, 2.2)	0.66
Hispanic	1.0 (−3.8, 5.8)	0.68	1.8 (−3.4, 6.9)	0.50	−0.5 (−3.3, 2.4)	0.74
Other	−10.0 (−19.5, −0.6)	**0.04**	−8.5 (−18.7, 1.7)	0.10	−1.9 (−7.6, 3.7)	0.50
Household income						
≥$80,000	Reference					
$40,000–$79,999	−4.4 (−8.8, −0.1)	**0.047**	−2.7 (−7.4, 2.0)	0.27	−1.8 (−4.4, 0.8)	0.18
<$40,000	−2.0 (−6.5, 2.5)	0.39	−0.9 (−5.8, 4.0)	0.71	−0.9 (−3.6, 1.8)	0.51
Prematurity						
No	Reference					
Yes	−0.7 (−4.8, 3.5)	0.75	−0.01 (−4.5, 4.5)	0.99	−0.6 (−3.0, 1.9)	0.65
Passive smoke exposure						
No	Reference					
Yes	−7.9 (−12.9, −3.0)	**0.002**	−9.3 (−14.6, −3.9)	**0.001**	1.0 (−1.9, 4.0)	0.50

Abbreviations: 25(OH)D, 25-hydroxyvitamin D; Q1, first (lowest) quintile of serum 25(OH)D; Q2, second quintile of serum 25(OH)D; Q3, third quintile of serum 25(OH)D; Q4, fourth quintile of serum 25(OH)D; Q5, fifth (highest) quintile of serum 25(OH)D. Bold denotes *p* < 0.05.

## Data Availability

The data that support the findings of this study are available on reasonable request from the corresponding author. The data are not publicly available due to privacy restrictions.

## Supplementary Materials

The following supporting information can be downloaded at: https://www.mdpi.com/article/10.3390/nu15102379/s1, Table S1: Characteristics of participants included and excluded from the cohort; Table S2: Association between quintiles of early childhood serum 25(OH)D and age 6 lung function using Global Lung Initiative references equations that incorporate race; Table S3: Association between early childhood quintiles of serum 25(OH)D and age 6 lung function with acceptable and reproducible maneuvers as per American Thoracic Society (ATS) criteria.
