# Supplementary material for: Association between Early Childhood Vitamin D Status and Age 6-Year Lung Function among Children with a History of Severe Bronchiolitis in Infancy
Source: Nutrients. 2023 May 19;15(10):2379. doi: 10.3390/nu15102379 (PMC10222498; doi:10.3390/nu15102379)
Supplement: Supplementary file 1 [file nutrients-15-02379-s001.zip › nutrients-2382605-supplementary.pdf]

Association between Early Childhood Vitamin D Status and Age 6-Year Lung Function among Children with a History of Severe Bronchiolitis in Infancy

George Doumat 1, Geneva D. Mehta 2, Jonathan M. Mansbach 3, Kohei Hasegawa 1 and Carlos A. Camargo, Jr. 1,*

1 Emergency Medicine Network, Massachusetts General Hospital, 125 Nashua Street, Suite 920, Boston, MA 02114, USA

2 Division of Allergy and Clinical Immunology, Brigham and Women's Hospital, Boston, MA 02115, USA

3 Division of General Pediatrics, Boston Children's Hospital, Boston, MA 02115, USA

* Correspondence: ccamargo@partners.org; Tel.: +617-726-5276

Table of contents:

- Supplement Table S1. Characteristics of participants included and excluded from the cohort

- Supplement Table S2. Association between quintiles of early childhood serum 25(OH)D and age 6-year lung function using Global Lung Initiative references equations that incorporate race

- Supplement Table S3. Association between early childhood quintiles of serum 25(OH)D and age 6-year lung function with acceptable and reproducible maneuvers as per American Thoracic Society (ATS) criteria

Supplement Table S1. Characteristics of participants included and excluded from the cohort

	**Included* (n=363)**	**Excluded (n=558)**	**P-value†**
**Age at enrollment in months, mean (SD)**	4.1 (2.9)	4.1 (3.0)	0.84
**Male sex, n(%)**	225 (62.0)	328 (58.8)	0.33
**Race/ethnicity, n(%)**			0.38
Non-Hispanic White	166 (45.7)	235 (42.1)	
Non-Hispanic Black	85 (23.4)	125 (22.4)	
Hispanic	102 (28.1)	172 (31.0)	
Other	10 (2.8)	25 (4.5)	
**Household income n=656 (%)**			0.79
⩾$80,000	88 (33.6)	123 (31.2)	
$40,000 – $79,999	67 (25.6)	108 (27.4)	
<$40,000	107 (40.8)	163 (41.4)	
**Premature birth, n(%)**	71 (19.6)	100 (17.9)	0.53
**Passive smoking, n(%)**	47 (34.1)	91 (16.3)	0.16

Abbreviation: SD, standard deviation.
* Analytical cohort
† Chi-squared test

Supplement Table S2. Association between quintiles of early childhood serum 25(OH)D and age 6-year lung function using Global Lung Initiative references equations that incorporate race

	FEV1 percent predicted		FVC percent predicted		FEV1 percent predicted /FVC percent predicted	
	Beta coefficient (95% CI)	P-value	Beta coefficient (95% CI)	P-value	Beta coefficient (95% CI)	P-value
**Serum 25(OH)D**						
Q5	Reference					
Q4	-4.80 (-9.50, -0.11)	**0.045**	-3.74 (-8.79, 1.30)	0.15	-1.04 (-3.82, 1.75)	0.46
Q3	-3.91 (-8.85, 1.04)	0.12	-4.43 (-9.73, 0.88)	0.10	0.17 (-2.76, 3.11)	0.90
Q2	-4.33 (-9.53, 0.86)	0.10	-4.20 (-9.77, 1.38)	0.14	-0.29 (-3.37, 2.79)	0.85
Q1	-5.83 (-11.16, -0.50)	**0.03**	-6.22 (-11.94, -0.49)	**0.03**	-0.03 (-3.19, 3.13)	0.99
**Household income**						
⩾ 80,000$	Reference					
40,000 - 79,999$	-3.58 (-7.95, 0.79)	0.11	-1.74 (-6.43, 2.95)	0.47	-1.76 (-4.35, 0.83)	0.18
< 40,000$	1.88 (-2.07, 5.84)	0.35	4.01 (-0.23, 8.26)	0.06	-1.57 (-3.91, 0.78)	0.19
**Prematurity**						
No	Reference					
Yes	0.28 (-3.83, 4.40)	0.89	1.17 (-3.25, 5.59)	0.6	-0.72 (-3.16, 1.72)	0.56
**Passive smoke exposure**						
No	Reference					
Yes	-9.08 (-13.97, -4.18)	**<0.001**	-10.81 (-16.07, -5.56)	**<0.001**	1.14 (-1.77, 4.04)	0.44

Abbreviations: 25(OH)D, 25-hydroxyvitamin D; Q1, first (lowest) quintile of serum 25(OH)D; Q2, second quintile of serum 25(OH)D; Q3, third quintile of serum 25(OH)D; Q4, fourth quintile of serum 25(OH)D; Q5, fifth (highest) quintile of serum 25(OH)D. Bold denotes *p*<0.05.

Supplement Table S3. Association between early childhood quintiles of serum 25(OH)D and age 6-year lung function with acceptable and reproducible maneuvers as per American Thoracic Society (ATS) criteria

	FEV1 percent predicted		FVC percent predicted		FEV1 percent predicted /FVC percent predicted	
N = 288	Beta coefficient (95% CI)	P-value	Beta coefficient (95% CI)	P-value	Beta coefficient (95% CI)	P-value
**Serum 25(OH)D**						
Q5	Reference					
Q4	-4.60 (-9.05, 0.23)	0.06	-4.12 (-9.47, 1.23)	0.13	-0.49 (-3.60, 2.61)	0.75
Q3	-4.83 (-9.91, 0.30)	0.07	-5.10 (-10.77, 0.57)	0.08	-0.04 (-3.29, 3.21)	0.98
Q2	-6.40 (-12.38 -0.41)	**0.04**	-5.70 (-12.32, 0.91)	0.09	-0.64 (-4.41, 3.12)	0.74
Q1	-6.62 (-12.37, -0.86)	**0.02**	-7.28 (-13.76, -0.91)	**0.03**	0.13 (-3.36, 3.63)	0.94
**Race/ethnicity**						
Non-Hispanic White	Reference					
Non-Hispanic Black	-13.61 (-18.78, -8.44)	**<0.001**	-14.45 (-20.17, -8.73)	**<0.001**	-0.30 (-3.62, 3.02)	0.86
Hispanic	-0.51 (-5.83, 4.81)	0.86	1.37 (-4.52, 7.26)	0.65	-1.46 (-4.89, 1.96)	0.40
Other	-7.82 (-17.51, 1.87)	0.11	-9.59 (-20.32, 1.13)	0.08	1.03 (-5.20, 7.27)	0.74
**Household income**						
⩾ 80,000$	Reference					
40,000 - 79,999$	-3.45 (-8.14, 1.24)	0.15	-1.04 (-6.24, 4.15)	0.69	-2.54 (-5.55, 0.46)	0.10
< 40,000$	0.38 (-4.46, 5.24)	0.88	0.96 (-4.41, 6.33)	0.72	-1.61 (-4.27, 1.03)	0.23
**Prematurity**						
No	Reference					
Yes	-0.91 (-4.53, 4.03)	0.68	0.55 (-4.16, 5.28)	0.82	-0.64 (-3.38, 2.10)	0.65
**Passive smoke exposure**						
No	Reference					
Yes	-9.46 (-14.80, -4.12)	**0.001**	-9.73 (-15.64, -3.82)	**0.001**	0.71 (-2.56, 4.00)	0.67

Abbreviations: 25(OH)D, 25-hydroxyvitamin D; Q1, first (lowest) quintile of serum 25(OH)D; Q2, second quintile of serum 25(OH)D; Q3, third quintile of serum 25(OH)D; Q4, fourth quintile of serum 25(OH)D; Q5, fifth (highest) quintile of serum 25(OH)D. Bold denotes $p<0.05$.
